# Cyanidin-3-glucoside Alleviates 4-Hydroxyhexenal-Induced NLRP3 Inflammasome Activation via JNK-c-Jun/AP-1 Pathway in Human Retinal Pigment Epithelial Cells

**DOI:** 10.1155/2018/5604610

**Published:** 2018-04-30

**Authors:** Xiaolu Jin, Chengtao Wang, Wei Wu, Tingting Liu, Baoping Ji, Feng Zhou

**Affiliations:** ^1^Beijing Advanced Innovation Center for Food Nutrition and Human Health, College of Food Science and Nutritional Engineering, China Agricultural University, Beijing 100083, China; ^2^Beijing Engineering and Technology Research Center of Food Additives, Beijing Technology & Business University, Beijing 100048, China; ^3^College of Engineering, China Agricultural University, Beijing 100083, China; ^4^Key Laboratory of Agricultural Big Data, Ministry of Agriculture, Beijing 100081, China

## Abstract

Recently, the NLRP3 inflammasome activation in the eyes has been known to be associated with the pathogenesis of age-related macular degeneration. The aim of this study was to investigate the protective effects of cyanidin-3-glucoside (C3G), an important anthocyanin with great potential for preventing eye diseases, against 4-hydroxyhexenal- (HHE-) induced inflammatory damages in human retinal pigment epithelial cells, ARPE-19. We noticed that C3G pretreatment to the ARPE-19 cells rescued HHE-induced antiproliferative effects. Cell apoptosis ratio induced by HHE was also decreased by C3G, measured by flow cytometry. The activation of NLRP3 inflammasome induced by HHE was found with increases of caspase-1 activity, proinflammatory cytokine releases (IL-1*β* and IL-18), and NLRP3 inflammasome-related gene expressions (NLRP3, IL-1*β*, IL-18, and caspase-1). The C3G showed potent inhibitive effects on these NLRP3 inflammasome activation hallmarks induced by HHE. Moreover, we noticed that the C3G's pretreatment leads to a delayed and a decreased JNK activation in HHE-challenged ARPE-19 cells. Finally, using a luciferase reporter gene assay system, we demonstrated that HHE-induced activation protein- (AP-) 1 transcription activity was abolished by C3G pretreatment in a dose-dependent manner. Taken together, these data showed that HHE leads to inflammatory damages to ARPE-19 cells while C3G has great protective effects, highlighting future potential applications of C3G against AMD-associated inflammation.

## 1. Introduction

Currently, age-related macular degeneration (AMD) is becoming the leading cause of elderly legal blindness in the world [1]. The Bruch's membrane, choroid, photoreceptors, and retinal pigment epithelium (RPE) are primarily affected by AMD, characterized with morphological and functional abnormalities. During AMD, the RPE becomes progressively dysfunctional and eventually degenerates, which leads to the death of photoreceptors and finally causes the loss of the visual function. The etiology and the molecular pathogenesis of AMD remain unclear, particularly for the atrophic subtype of this disease. Experimental and clinical studies recently identified that the intense parainflammation to the RPE is a contributing factor in the development of AMD [2, 3]. Therefore, recent studies have focused on the inflammasome signaling pathway, which leads to the development of clinically relevant novel therapeutic strategies [4, 5].

Inflammation is involved in innate immune response against exogenous pathogens and is considered as the first line of defense against pathogenic microbes as well as cellular stress [6]. The activation of inflammasome is important for transducing these “danger” signals for triggering inflammation. Inflammasomes lead to the activation of caspase-1 and the following cleavage of IL-1*β* and IL-18, the two major proinflammatory cytokines. A series of inflammasome complexes have been clarified, and each of them was identified with a unique pattern recognition receptor (PRR) and activation triggers. Among these complexes, NLRP3 inflammasome is the best characterized one, which is consisted by NLRP3, apoptosis-associated speck-like protein, serine-threonine kinase NEK7, and procaspase-1 [7]. The NLRP3 inflammasome is activated in a 2-step process. First, NF-*κ*B signaling is induced through pathogen-associated molecular patterns (PAMPs) or danger-associated molecular pattern- (DAMP-) mediated activation of TLR4 or TNFR, which result in the increased expression of NLRP3, pro-IL-1*β*, and pro-IL-18. Next, inflammatory microenvironmental factors (whole pathogens, PAMPs/DAMPs, or endogenous factors) induced an indirect activation of NLRP3 and induced the activation of caspase-1 which eventually leads to the maturation and secretion of IL-1*β* and IL-18 [8]. The NLRP3 inflammasome has been found to be present in samples from AMD patients [9]. Several compounds associated with AMD have been shown to activate the NLRP3 inflammasome, like the complement component C5a and AMD including drusen components including C1q and amyloid-*β* [3], the lipofuscin component *N*-retinylidene-*N*-retinyl-ethanolamine (A2E), and the lipid peroxidation product 4-hydroxynonenal (HNE) [10]. Previous studies also noticed that photooxidative damage to the RPE-accumulated lipofuscin will activate the NLRP3 inflammasome [11].

Current thinking suggests that RPE cells are constantly exposed to lipid peroxidation. In the retinal tissues, free radicals can directly attack critical biomolecules including polyunsaturated fatty acids (PUFAs) and lead to its degradation into oxidized products, including aldehydes. Among these hazardous lipid products, 4-hydroxynonenal (4-HNE) and 4-hydroxyhexenal (4-HHE) are the most investigated unsaturated aldehydes. 4-HNE is derived from n-6 PUFAs, like linoleic acid and arachidonic acid, while 4-HHE is formed from n-3 PUFAs such as docosahexaenoic acid (DHA), eicosapentaenoic acid (EPA), and linolenic acid [12]. These three major unsaturated fatty acids reached about 50%, 10%, and 8% of the total fatty acids, respectively, in human retina [13]. Due to the structure similarities of 4-HNE and 4-HHE, these two aldehydes showed a number of cytotoxic effects, including inhibition on selective enzyme activity, induction on cell cycle arrest, and cell apoptosis [14]. Recent study also highlighted the roles of 4-HNE during NLRP3 inflammasome activation in human retinal pigment epithelium cells, ARPE-19 [11]. In this work, the potential damages caused by daily visible light exposure on retinal UFAs were evaluated via a simulated *in vitro* model [13]. Moreover, our previous study showed that the lipid peroxidation of DHA affects the physiological health of the retina cells [13, 15]. Nevertheless, as the major lipid oxidized products from DHA, whether 4-HHE has a proinflammatory effect is still unknown.

Anthocyanins are strong antioxidants which have been evidenced to be beneficial for vision [16]. Cyanidin-3-glucoside (C3G) is an important anthocyanin found in purple fruits and rice with great beneficial potentials for preventing eye diseases [17, 18]. It has been examined with respect to different steps in the visual signal transduction process. Previous studies noticed that it inhibits the photooxidation of RPE cells via facilitating the regeneration of rhodopsin in rod photoreceptors [19, 20]. Moreover, C3G has been reported to regulate the visual signal transduction. For example, in rod outer segments, C3G inhibited the activation of the G-protein induced by light exposure via metarhodopsin II [21]. Our previous studies confirmed that the retinal protective activity of C3G against light-induced retinal injury was confirmed *in vivo* while the underlying mechanisms remain unclear [13, 18, 22]. In this article, we aim to know whether 4-HHE might induce activation of inflammasome signaling in ARPE-19 cells and furthermore that the polyphenol compound, C3G, is able to protect RPE cells against inflammatory damage.

## 2. Material and Methods

### 2.1. Cell Culture and Treatment

Human retinal pigment epithelium cells, ARPE-19, were obtained from the ATCC and cultured in DMEM/F12 medium (Gibco BRL, Grand Island, NY) with 10% fetal bovine serum (FBS; Thermo Fisher Scientific, Waltham, MA) in a humidified incubator at 37°C in 5% CO_2_, supplemented with 100 U/mL penicillin and 100 *μ*g/mL streptomycin (Sigma-Aldrich, St. Louis, MO, USA). For cell treatment, cells were washed with prewarmed PBS then the complete culture medium was replaced by FBS-free medium. To induce inflammatory damage, 4-hydroxyhexenal (Cayman Chemical, Ann Arbor, MI, USA) which were dissolved in ethanol were challenged to the cells for 24 h. After determining the optimal dosage of cyanidin-3-glucoside (Yuanye Biological Technology Co. Ltd., Shanghai, China), which was dissolved in DMSO, cells were pretreated with C3G for 2 h before 4-HHE stimulations as indicated in the experiments. All studies were performed using 70–80% confluent cells before treatment.

### 2.2. Cell Viability Assays

To measure the cytotoxicity of HNE and C3G treatments, cell medium samples were analyzed for CCK-8 and lactate dehydrogenase (LDH) enzyme activity. After designed treatments, 10 *μ*L of CCK-8 solution (Dojindo, Kumamoto, Japan) was added into 96-well plates and OD 450 nm was measured using a microplate reader (M5, MD, USA). The LDH activity was also determined by a commercial kit (Beyotime, Haimen, China) according to the manufacturer's instructions.

### 2.3. Cell Apoptosis Measurement

ARPE-19 cell apoptosis was assessed using an Annexin V-FITC Apoptosis Detection Kit (BD Biosciences Pharmingen, San Diego, CA, USA). Briefly, after indicated treatments, cells were washed and harvested then resuspended in 100 *μ*L Annexin Binding Buffer. Cells were stained with Annexin V-FITC (5 *μ*L) and propidium iodide (PI, 1 *μ*L) solution. The flow cytometric analysis was performed using a FACScan flow cytometer (Becton Dickinson, Franklin Lakes, NJ, USA).

Cellular caspase-1 activity was determined using a commercial kit based on colorimetric assay according to the manufacturer's instructions (Beyotime, Haimen, China). Briefly, cells were treated as designed then lysed and centrifuged at 10,000 ×g for 1 min at room temperature. Total cytosolic protein (50 *μ*g) was incubated with acetyl-Tyr-Val-Ala-Asp p-nitroaniline (Ac-YVAD-pNA, 20 nM) for 2 h at 37°C. The absorbance values were measured at 405 nm using a microplate reader (M5, MD, USA).

### 2.4. Enzyme-Linked Immunosorbent Assay (ELISA)

Cell culture supernatants from treated cells were collected, and cytokines were measured by using commercial IL-1*β* and IL-18 ELISA kits (R&D Systems, Minneapolis, MN, USA), following the manufacturer's instructions.

### 2.5. Quantitative Real-Time PCR

According to the instructions from the manufacturer, cellular RNA was extracted using the RNeasy® Plus Mini Kit (Qiagen, Valencia, CA, USA). Reverse transcription was carried out using the PrimeScript RT Reagent Kit (TaKaRa, Dalian, China). Real-time PCR was performed with the 7500c Real-time PCR Detection System (Applied Biosystems, Carlsbad, CA, USA) with SYBR Premix Ex Taq (TaKaRa) following the manufacturer's instructions. Primers were designed to flank introns with the Primer 5 software (Premier Biosoft, Palo Alto, CA, USA), and the primers sets were as follows: *GAPDH*, 5′-AGGGATGATGTTCTGGAGAG-3′ (F) and 5′-TCAAGATCATCAGCAATGCC-3′ (R); *NLRP3*, 5′-TCGGAGATTGTGGTTGGG-3′ (F) and 5′-GGGCGTTGTCACTCAGGT-3′ (R); *IL-1β*, 5′-CTAAACAGATGAAGTGCTCCTTCC-3′ (F) and 5′-CACATAAGCCTCGTTATCCCA-3′ (R); *IL-18*, 5′-ATCAGGATCCTTTGGCAAGCTTGAATCTAAATTATC-3′ (F) and 5′-ATAGGTCGACTTCGTTTTGAACAGTGAACATTATAG-3′ (R) [23]; and *caspase-1*, 5′-TGGTCTTGTGACTTGGAGGA-3′ (F) and 5′-TGGCTTCTTATTGGCACGAT-3′ (R) [24]. Data were calculated using equation 2^−ΔΔCt^ and normalized to the expression of the housekeeping gene *(GAPDH)* and expressed as fold change against the control group.

### 2.6. Western Blot Analysis

Cellular proteins were lysed, and equal amounts of protein (20 *μ*g) were separated by SDS-PAGE and then transferred to PVDF membranes, which were incubated with primary antibodies, phospho-JNK1/2 (Thr183/Tyr185) and *β*-tubulin (Cell Signaling Technology, Danvers, MA; Abcam, Cambridge, UK). Bound antibody complexes were visualized using NBT/BCIP solution with color development buffer.

### 2.7. Luciferase Reporter Assay

ARPE-19 cells were seeded in 6 cm plates and transfected with 3 *μ*g of pGL3-AP1 vector. After transfection, cells were separated into a 24-well plate followed by treatment with the indicated concentrations of C3G for 2 h before the addition of HHE for another 24 h. Luciferase activity was measured using the dual luciferase assay system (Promega, Madison, WI, USA) according to the manufacturer's instructions. A Renilla luciferase-expressing vector (pRL-TK) was cotransfected with firefly luciferase and used as the internal control. Promoter activity is presented as multiples of the control.

### 2.8. Statistical Analyses

Quantitative data are presented as the arithmetic mean *±* standard error of the mean (SEM) for each treatment group. The effect of treatments was determined by one-way analysis of variance (ANOVA), and differences between treatments were analyzed post hoc by Tukey's honest significant difference test. A *P* value less than 0.05 was considered statistically significant. All statistical tests were performed using SPSS version 17.0 (SPSS Inc., Chicago, IL).

## 3. Results

### 3.1. C3G Inhibited HHE-Induced Antiproliferative Effect via Suppressing RPE Cell Apoptosis

**Figure 1:**
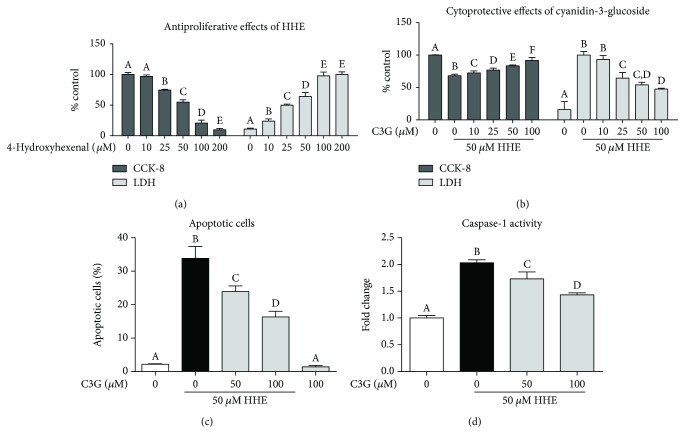
C3G inhibited HHE-induced antiproliferative effect via suppressing RPE cell apoptosis. (a) HHE showed antiproliferative effects in ARPE-19 cells. ARPE-19 cells were incubated with various dosages of HHE for 24 h in serum-free culture mediums, and then the cell viability was measured by cell counting kit- (CCK-) 8 and LDH (lactate dehydrogenase) tests as described in Materials and Methods. (b) C3G showed protective effects against HHE-induced antiproliferative effects in ARPE-19 cells. Cells are incubated with or without C3G for 2 h then challenged with HHE for 24 h in serum-free culture mediums. The cell viability was measured by CCK-8 assay and LDH tests. (c) Cell apoptosis induced by HHE was inhibited by C3G. C3Gs (50, 100 *μ*M) were pretreated to the ARPE-19 cells for 2 h, and then HHE was challenged for 24 h in serum-free culture mediums. ARPE-19 cell apoptosis was determined by Annexin V/PI staining. Data reported as percentage of Annexin V-positive cells (early and late apoptotic cells). (d) Caspase-1 activity was measured by a colorimetric assay. Bars with different letters are significantly different from each other (*P* < 0.05, *n* = 6).

As shown in Figure 1(a), various concentrations of HHE (10 to 200 *μ*M) were applied to the ARPR-19 cells and we noticed notable antiproliferative effects followed by a 24 h HHE treatment, with significant decreased of reduction in the viability, and dramatical LDH enzyme releases (25, 50, 100, and 200 *μ*M; *P* < 0.05 and *P* < 0.01, resp.). Moreover, to explore whether C3G has any protective effects against HHE-induced antiproliferative effect, we tested various concentrations of C3G and pretreated them to the cells 2 h before the HHE challenge. As shown in Figure 1(b), when C3Gs (25, 50, and 100 *μ*M) were added to the cell cultures, the compromised metabolic activity of cells by HHE were improved. In addition, HHE-induced LDH releases were significantly diminished by C3G. On a molecular level, we noticed that cell apoptosis ratio induced by HHE was decreased by C3G, measured by flow cytometry (Figure 1(c)). Furthermore, caspase-1 activity was increased by 2.03-fold in ARPE-19 cells after 24 h HHE treatment, and 50 and 100 *μ*M C3G treatment to the HHE-challenged cells reduced the caspase-1 activity significantly (Figure 1(d)).

### 3.2. C3G Reduced the Production of IL-1*β* and IL-18 Induced by HHE in RPE Cells

**Figure 2:**
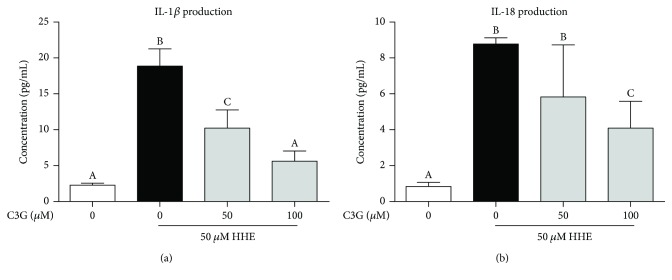
C3G reduced the production of IL-1*β* and IL-18 induced by HHE in RPE cells. C3Gs (50, 100 *μ*M) were pretreated to the ARPE-19 cells for 2 h, and then HHE was challenged for 24 h in serum-free culture mediums. Cell culture mediums were collected, and the production of IL-1*β* (a) and IL-18 (b) is determined by ELISA. Bars with different letters are significantly different from each other (*P* < 0.05, *n* = 6).

We hypothesized that HHE might lead to inflammatory damages in ARPE-19 cells and C3G might exert an anti-inflammatory effect. Therefore, we next tested proinflammatory cytokine releases in HHE- and C3G-treated ARPE-19 cells. As shown in Figure 2, 50 *μ*M HHE for 24 h leads to dramatical increases of IL-1*β* and IL-18. Strikingly, compared with the HHE-treated control, C3G (100 *μ*M) pretreatment to ARPE-19 cells for 2 h leads to a decrease of IL-1*β* and IL-18 for 66% and 45%, respectively.

### 3.3. C3G Inhibited the Activation of NLRP3 Inflammasome Induced by HHE in RPE Cells

**Figure 3:**
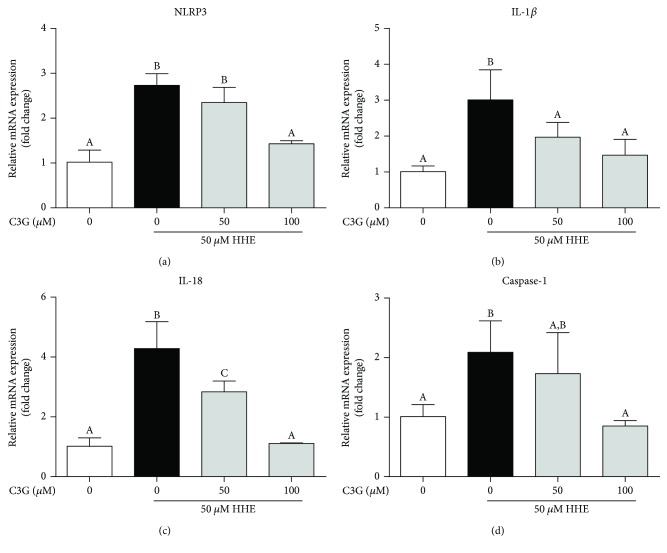
C3G inhibited the activation of NLRP3 inflammasome induced by HHE in RPE cells. C3Gs (50, 100 *μ*M) were pretreated to the ARPE-19 cells for 2 h, and then HHE was challenged for 6 h in serum-free culture mediums. Total RNA was collected and relative NLRP3, IL-1*β*, IL-18, and caspase-1 mRNA level are measured by qRT-PCR.

To evaluate the roles of the NLRP3 inflammasome complex during C3G-induced protective effects, ARPE-19 cells were pretreated with C3G and challenged with HHE. Several key NLRP3 inflammasome components, including NLRP3, IL-1*β*, IL-18, and caspase-1 mRNA levels, are measured by qRT-PCR. As shown in Figure 3(a), C3G significantly decreased the HHE-induced mRNA level of NLRP3 in ARPE-19 cells. Activation of NLRP3 inflammasome was also notified by upregulations of IL-1*β*, IL-18, and caspase-1. C3G showed potent inhibitive effects on decreasing these gene expressions in HHE-primed ARPE-19 cells (Figure 3(b)–3(d)).

### 3.4. JNK Activation Plays a Critical Role during C3G's Protective Effects against HHE-Induced Cell Death

**Figure 4:**
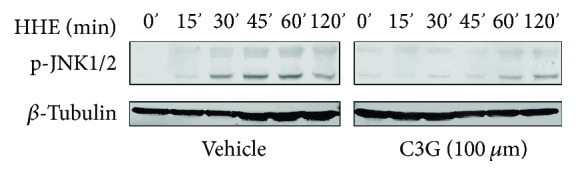
C3G blunting HHE-induced JNK activation in RPE cells. Confluent ARPE-19 cells were pretreated with 100 *μ*M C3G or vehicle control (DMSO) for 2 h. Then, HHE (50 *μ*M) was challenged to the cells for various time periods. Proteins were collected and analyzed using Western blotting.

It was our interest to reveal which signal pathways are involved in the C3G's protective effects to ARPE-19 cells challenged by HHE. ARPE-19 cells were challenged with HHE for various time periods and found that JNK were activated after 30 min treatment of HHE (Figure 4(a)). Indeed, C3G's pretreatment leads to a delay and a decrease on the JNK activation (Figure 4(b)).

### 3.5. Effects of C3G on AP-1 Activity during HHE-Induced NLRP3 Inflammasome Activation

**Figure 5:**
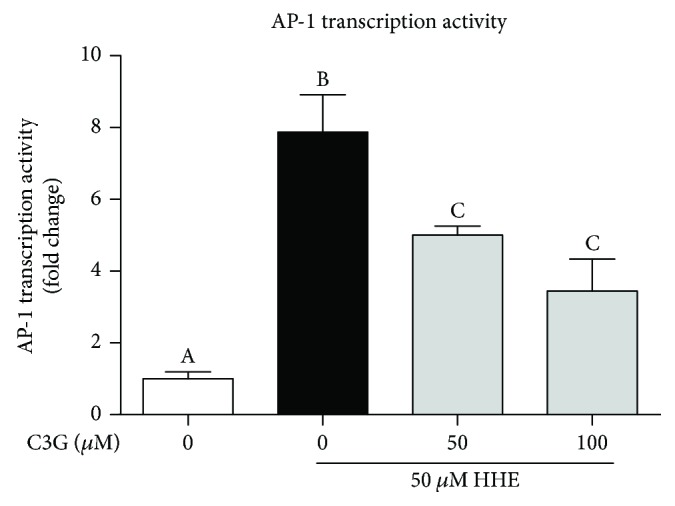
Effects of C3G on AP-1 activity during HHE-induced NLRP3 inflammasome activation. ARPE-19 cells were transfected with plasmid with AP-1 response element (p pAP1-TA-luc) and a Renilla luciferase-expressing vector (pRL-TK) for 24 h. Then, cells were pretreated with C3G for 2 h and then HHE (50 *μ*M) was challenged for 12 h in serum-free culture mediums. The promoter activities of AP-1 were analyzed with a luciferase reporter assay. Values are expressed as the mean ± SD from three independent experiments. Bars with different letters are significantly different from each other (*P* < 0.05).

Accumulating studies suggest that the activation of the transcription factor activator protein 1 (AP-1) is essential for the transcriptional regulation of inflammasome-related genes. The effects of AP-1 transcription activity were measured by a dual luciferase reporter gene assay system (Figure 5). The activities of AP-1 transcription factor were significantly increased by HHE and C3G dose dependently abolished AP-1 transcription activity in ARPE-19 cells.

## 4. Discussion

The lipid peroxidation product 4-hydroxyhexenal (HHE, also known as *trans*-4-hydroxy-2-hexenal) is an important biochemical mediator originated from n-3 PUFA [14]. Nevertheless, compared with 4-hydroxynonenal (HNE), another major unsaturated aldehyde oxidation product of n-3 PUFA, studies relating to its physiologic/pathological importance are quite limited. Here, we found for the first time that HHE induces cytotoxicity and activates NLRP3 inflammasome in human retinal pigment epithelium cells. Importantly, cyanidin-3-glucoside, a major anthocyanin with great nutritional supplement potentials for preventing retinal degenerative diseases, was able to protect ARPE-19 cells from HHE-induced inflammatory damages.

During n-3 PUFA peroxidation, several aldehyde products were generated, and at least 15 aldehydes were identified, including HNE, HHE, glyoxal, malondialdehyde, and 2-pentenal [14]. Like HNE, comparable cytotoxicity was investigated by researchers in assorted cell types, including primary cells as well as immortalized cell lines. Regarding retinal cells, RPE-19 epithelial cells are more sensitive to toxicity by HNE than HHE, which differs from neuronal system [25]. In our hands, the toxic concentration of HHE (50 *μ*M) to RPE-19 cell viability is comparable to previous studies [26, 27]. In addition, the proapoptotic effects by HHE have also been reported in endothelial cells [28], neuron cells [25], and lens epithelial cells [26, 27]. Despite the activation on caspases of HNE has been noticed by several previous reports, its analog, HHE also showed similar induction effects on caspase-1 [11, 27].

It is agreed that caspase-1 is activated by certain inflammasomes, including NLRP1, NLRP3, NLR family CARD-containing 4 (NLRC4), and absent in melanoma 2 (AIM2). Among them, the NLRP3 inflammasome is the best characterized one, which is consisted by a multiprotein complex composed of NLRP3, apoptosis-associated speck-like (ASC) protein, which contains an adaptor for caspase recruitment domain, and procaspase-1 [29]. Selected activators/pathogen stimuli lead to the NLRP3 inflammasome activation, which leads to the molecular modification of procaspase-1, then cleaving it into caspase-1 and thereby mediating the proinflammatory cytokines, IL-1*β*, and IL-18 maturation and secretion from its precursors. During AMD, a variety of substances leads to the inflammasome activation in the retinal epithelium, including the drusen components [24], the lipofuscin component *N*-retinylidene-*N*-retinyl-ethanolamine (A2E) [3], and the lipid peroxidation product HNE [11]. In our hands, we noticed that HHE treatment also leads to substantial increases of IL-1*β* and IL-18, which fit well in the classical function of inflammasome pathway.

*In vivo* and *in vitro* reports have demonstrated that a dietary supplement of anthocyanin compounds can protect RPE and photoreceptor cells from light and inflammatory damage [30, 31]. Our previous studies showed the beneficial properties of bilberry-derived polyphenolic compounds (quercetin, anthocyanins, protocatechuic acid, ferulic acid, and chlorogenic acid) against ROS production and inflammatory damages induced by photooxidation in the retina [18, 32]. C3G is one of the most abundant anthocyanins in the edible parts of plants [18]. Recently, it has gained wide attention for its versatile beneficial effects, like antioxidant [32], anti-inflammatory [33], neuroprotective [34], cardiovascular protective properties, and others [35]. In the eyes, it is a strong anthocyanin which alleviated light-induced retinal oxidative stress, inflammation, and apoptosis via activation of Nrf2/HO-1 pathway and inactivation of NF-*κ*B in pigmented rabbits [18]. Recently, a group of researchers noticed that quercetin protects ARPE-19 cells from HNE-induced cytotoxicity and inflammation [36]. Even though there are no direct studies using HHE-induced ARPE-19 cell inflammatory damage, the anti-inflammatory effects of C3G are consistent with these studies.

Besides the evidence that showed a drop of HHE-induced NLRP3 inflammasome activation by C3G, we also studied the signaling behind these effects. It has been known that reactive aldehydes, such as HHE and HNE, have been implicated as inducers which activate cellular stress signaling pathways and integrate with additional signals in response for extracellular stimuli [37]. HNE is able to induce both intracellular signaling and intercellular signaling, including NF-*κ*B, Nrf-2 protein kinase C, and MAPK signals. Nevertheless, such studies relating to HHE and cellular signaling are rare compared with HNE. In endothelial cells, HHE was found to induce NF-*κ*B activation and MAPK activation, which is associated with oxidative stress [38, 39]. In a model for nonalcoholic fatty liver disease (NAFLD), the persistent JNK activation was noticed by HNE which links to oxidative stress and hepatocyte cell death [40]. During HNE-induced muscle cell apoptosis, HNE leads to the activation of JNK which mediated the antiapoptotic protein inactivation and caspase activation [41]. Pretreatment of well-known antioxidants, like resveratrol and piceatannol, suppresses JNK phosphorylation and subsequently blunts the AP-1 signaling [42, 43]. Given the electrophilic nature of *α*,*β*-unsaturated aldehydes like HNE [14], it is not surprising that HNE activates JNK and AP-1 signaling. Similar to resveratrol, C3G also showed potent regulating effects on these cellular signaling pathways; however, given that inflammasome activation turns on the number of other signaling cascades, the effects of C3G on these cascades need to be clarified in the future.

## 5. Conclusion

Taken together, our study clearly showed that the cytotoxic effects by HHE in APRE-19 cells were significantly rescued by C3G pretreatment. We also demonstrated for the first time that C3G inhibited HHE-induced releases of proinflammatory cytokines, IL-1*β*, and IL-18 and blunted NLRP3 inflammasome activation. Moreover, we also showed that JNK-c-Jun/AP-1 pathway activation by HHE is regulated by C3G, which shed a light for future potential treatment against AMD-associated inflammation.
